# Reisomerization of retinal represents a molecular switch mediating Na^+^ uptake and release by a bacterial sodium-pumping rhodopsin

**DOI:** 10.1016/j.jbc.2022.102366

**Published:** 2022-08-11

**Authors:** Tomotsumi Fujisawa, Kouta Kinoue, Ryouhei Seike, Takashi Kikukawa, Masashi Unno

**Affiliations:** 1 Department of Chemistry and Applied Chemistry, Faculty of Science and Engineering, Saga University, Saga, Japan; 2 Faculty of Advanced Life Science, Hokkaido University, Sapporo, Japan

**Keywords:** vibrational spectroscopy, low-temperature spectroscopy, microbial rhodopsin, membrane transporter protein, photoreceptor, BR, bacteriorhodopsin, HOOP, hydrogen out-of-plane, *Ia*NaR, NaR from *Indibacter alkaliphilus*, LED, light emitting diode, NaR, sodium-pumping rhodopsin, RSB, retinal schiff base

## Abstract

Sodium-pumping rhodopsins (NaRs) are membrane transporters that utilize light energy to pump Na^+^ across the cellular membrane. Within the NaRs, the retinal Schiff base chromophore absorbs light, and a photochemically induced transient state, referred to as the “O intermediate”, performs both the uptake and release of Na^+^. However, the structure of the O intermediate remains unclear. Here, we used time-resolved cryo-Raman spectroscopy under preresonance conditions to study the structure of the retinal chromophore in the O intermediate of an NaR from the bacterium *Indibacter alkaliphilus*. We observed two O intermediates, termed O1 and O2, having distinct chromophore structures. We show O1 displays a distorted 13-*cis* chromophore, while O2 contains a distorted all-*trans* structure. This finding indicated that the uptake and release of Na^+^ are achieved not by a single O intermediate but by two sequential O intermediates that are toggled via isomerization of the retinal chromophore. These results provide crucial structural insight into the unidirectional Na^+^ transport mediated by the chromophore-binding pocket of NaRs.

The movements of substances into or out of cells are achieved via transmembrane proteins, known as membrane transporters. The polar molecules and charged ions, for instance, are prevented from entering the hydrophobic interior of cellular membranes. For these substances, the only way to traverse the cellular membrane is via a route provided by the membrane transporters. This transportation involves the uptake of a substrate from one side of the membrane and its release to the other side. These two steps are believed to require a distinct structural change of the protein to switch the accessibilities of the substrate from one side and the other (1). However, this structural change of the transporting process has not been observed for most proteins.

Discovered in 2013, sodium-pumping rhodopsins (NaRs) are unique photoreceptive membrane transporters that function as light-driven Na^+^ pumps (2). NaRs are rhodopsin-like photoreceptors that have seven transmembrane helices (A, B, …, and G) as shown in Fig. 1*A*, where the retinal molecule is bound to the lysine residue on the helix G via a Schiff base linkage. This retinal Schiff base (RSB) chromophore is protonated and has the all-*trans* form before absorption of a photon (Fig. 1*B*). Then, photoabsorption induces the isomerization of the chromophore into the 13-*cis* form, and the subsequent chemical changes lead to Na^+^ transport from the cytoplasm to the extracellular side. This photoreaction is a cyclic process known as a photocycle and it produces multiple intermediate states typically expressed as NaR + *hν* → K → L/M → O → NaR (2, 3, 4). The K intermediate is the first state formed with a distorted 13-*cis* form (5). The next state, the L intermediate, is generated by structural relaxation of the distorted chromophore (6). This L intermediate is in equilibrium with the M intermediate, where the Schiff base moiety of the chromophore is deprotonated. Then, the recovery to the dark state is mediated by the O intermediate, which finally transports Na^+^ across the membrane.

**Figure 1:**
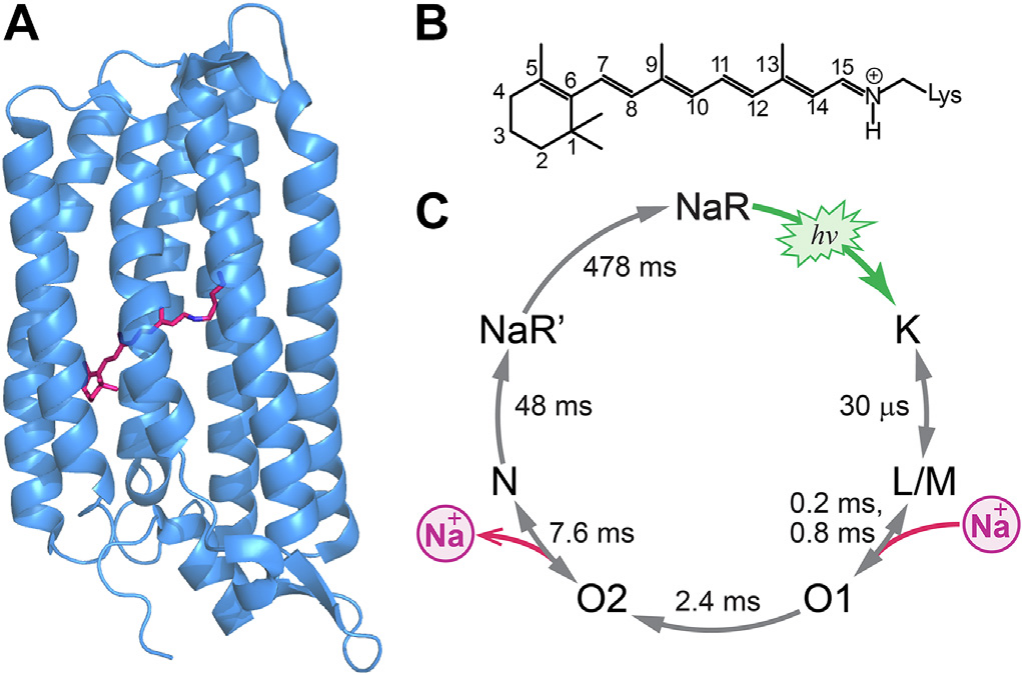
**Structure and photocycle of NaR.** (A), crystal structure (PDB entry: 6REW). (B), chemical structure of the retinal Schiff base chromophore. (C), photocycle of *Ia*NaR from ref. (14). The time constants shown with the photointermediates are obtained at 298 K in Tris–HCl buffer (pH 8) with 50 mM NaCl. NaR, sodium-pumping rhodopsin; *Ia*NaR, NaR from *Indibacter alkaliphilus*.

Of these multiple photointermediates, the O intermediate is recognized to play the most crucial role in membrane transport of Na^+^ (7). So far, most studies assumed a single state for the O intermediate. In an early transient absorption study, the redshifted absorption band was observed in the millisecond region, and this band was attributed to the O intermediate (2). Then, FTIR, Raman, and NMR studies of NaRs were conducted and reported the single O-intermediate spectrum (8, 9, 10, 11). However, recent studies came to find the evidences of multiple O intermediates. The X-ray crystallographic studies of an NaR from *Krokinobacter eikastus* (KR2), which were independently carried out by Skopintsev *et al*. (12) and Kovalev *et al*. (13), reported the different structures of the O intermediate. The former study by Skopintsev *et al*. also measured the time-resolved UV-visible and IR absorption spectra to report two O intermediates from a global fit analysis. We recently measured the time-resolved absorption spectra of a NaR from *Indibacter alkaliphilus* (*Ia*NaR) and revealed the clear presence of two O intermediates, O1 and O2, exhibiting distinct spectra and kinetics (14). The photocycle of *Ia*NaR is shown in Fig. 1*C*, where O1 (lifetime: 2.4 ms) has an absorption maximum (*λ*_max_) at 596 nm, while the subsequently formed O2 (lifetime: 7.6 ms) has a *λ*_max_ at 570 nm. After O2, the dark state recovers through N and NaR’. The detailed spectral analysis indicated that the O1 → O2 process was irreversible. On the basis of the time-resolved membrane voltage measurement (7), O1 and O2 were shown to be responsible for the Na^+^ uptake and release, respectively. However, the molecular mechanism based on the structures of these O intermediates remains an open question.

In this study, we carried out the time-resolved Raman measurement of *Ia*NaR at 268 K under preresonance condition to identify the structures of RSB chromophore in the O intermediates. This cryo-Raman method allowed us to accumulate the specific photointermediates by controlling the temperature and observe their structural transitions based on the time-dependent Raman spectra. Because of the sufficient accumulation of the photointermediates at low temperatures, we can apply the near-IR Raman excitation to measure the spectra without the fluorescence interference or the unfavorable side products, which often complicate the interpretation of the time-resolved resonance Raman spectra (15, 16). We successfully observed the vibrational spectra of O1, O2, and N. The spectral features indicated that O1 and O2 have the 13-*cis* and all-*trans* forms of the RSB chromophore, respectively. This result shows that the reisomerization of the chromophore during the O1 → O2 process irreversibly switches the Na^+^ uptake and release states of *Ia*NaR to achieve the unidirectional Na^+^ transport.

## Results

**Figure 2:**
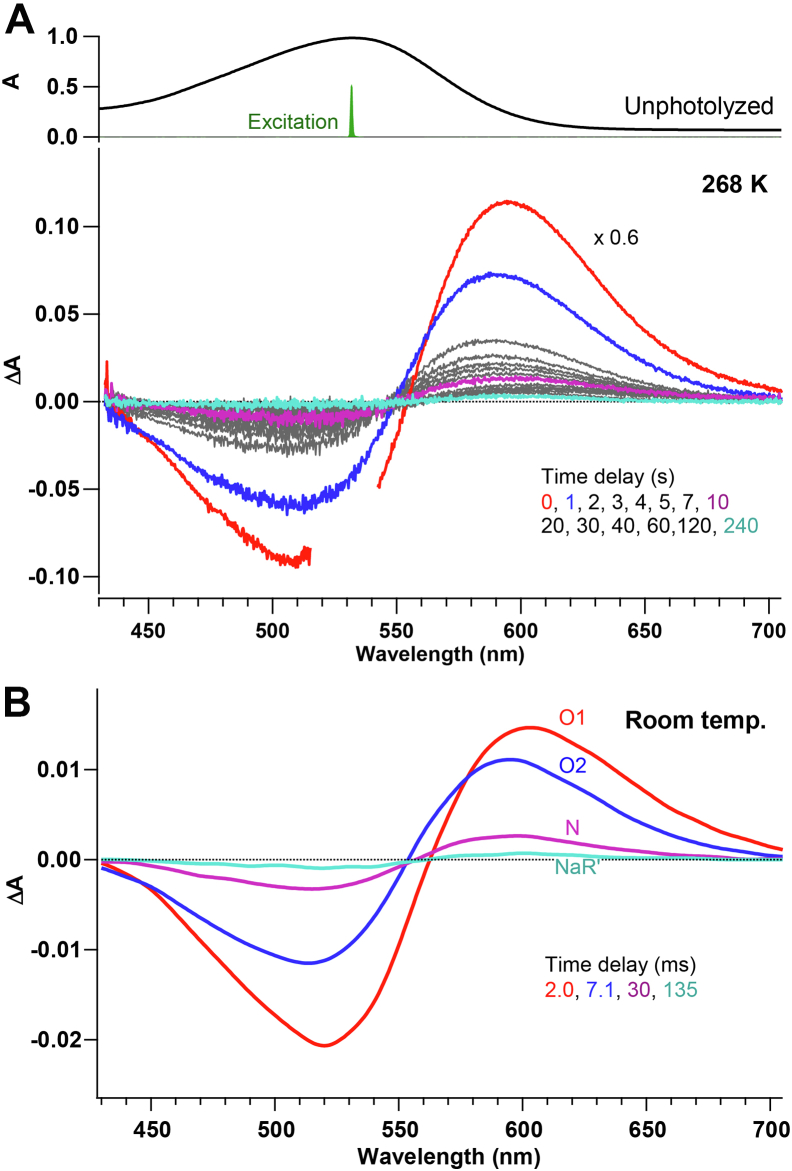
**Photoreaction of*****Ia*****NaR at 268 K and room temperature.** (A), time-resolved absorption spectra of *Ia*NaR at 268 K. The time resolution is 1 s. See Experimental procedures for details. (B), time-resolved absorption spectra of *Ia*NaR (with 50 mM NaCl) measured in our previous nanosecond flash photolysis study (14) at room temperature. The spectra are plotted at selected time points—namely, 2.0 ms (*red*), 7.1 ms (*blue*), 30 ms (*purple*), and 135 ms (*cyan*)—and O1, O2, N, and NaR′ make the major contributions to these four spectra, respectively (14). NaR, sodium-pumping rhodopsin; *Ia*NaR, NaR from *Indibacter alkaliphilus*.

We first describe the photoreaction of *Ia*NaR at 268 K based on the time-resolved absorption data. Fig. 2*A* shows the time-resolved absorption spectra at 268 K. In this measurement, the *Ia*NaR sample was irradiated by green light (at 532 nm) for 1 s and the light-induced absorbance changes were measured at the delay times (Δ*T’*s) up to 240 s with the time resolution of 1 s. The spectrum at Δ*T* = 0 s was measured at the photostationary state under continuous irradiation. As seen from the figure, the green light irradiation produced the redshifted absorption component, which decayed in a few hundred seconds. The spectra also exhibited the small shifts with Δ*T*. The time-resolved spectra showed an ∼10-nm blueshift from Δ*T* = 0 to 1 s and it then slightly redshifted afterward. Previously, we carried out the nanosecond flash photolysis of *Ia*NaR at room temperature (14) and observed the redshifted intermediates in the millisecond region as shown in Fig. 2*B*. The detailed kinetic analysis revealed that the millisecond process includes the four redshifted intermediates referred to as O1, O2, N, and NaR’ (Fig. 1*C*). In Fig. 2*B*, the spectral change from 2 to 7 ms is mainly caused by the O1 → O2 process. The spectral change after 7 ms is then attributed to the subsequent process of O2 → N → NaR’. The former spectral change at room temperature shows close similarity with that from 0 to 1 s at 268 K, and the latter spectral change corresponds to that after 1 s at 268 K. Thus, the time-resolved absorption data at 268 K confirms the production of the O intermediates and the subsequent transient states with their prolonged lifetimes.

**Figure 3:**
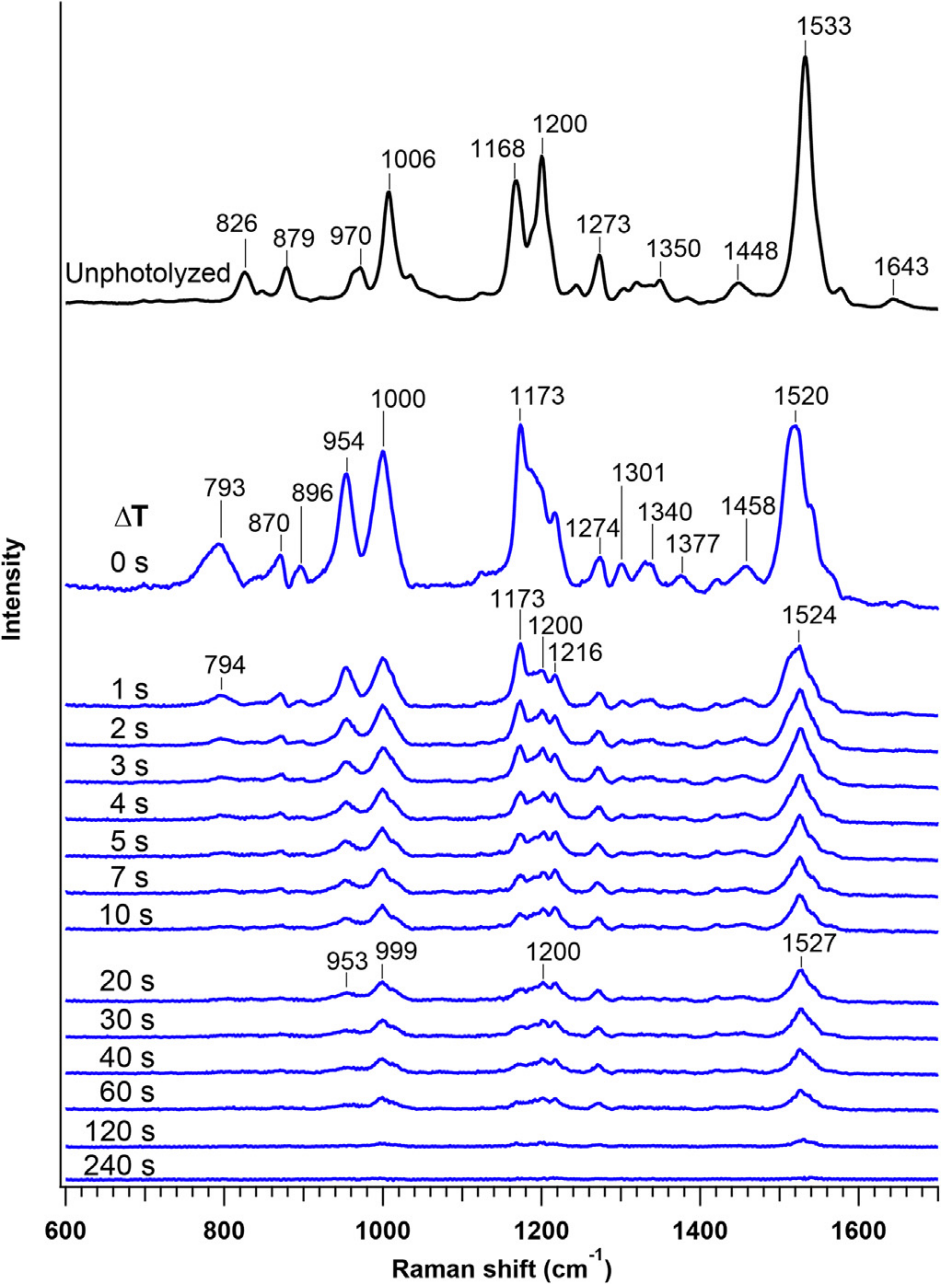
**Time-resolved Raman spectra of *Ia*NaR at 268 K.** The spectra were measured using a 785-nm probe after photoirradiation by 532-nm light. The spectrum of the unphotolyzed state (*top*) is shown for comparison. See Experimental procedures for details. *Ia*NaR, NaR from *Indibacter alkaliphilus*.

Fig. 3 shows the time-resolved Raman spectra at 268 K in comparison to the spectrum for the unphotolyzed state. These spectra were measured by 785-nm excitation and obtained in the same way as we did for the time-resolved absorption measurement; the *Ia*NaR sample was photoirradiated at 532 nm for 1 s and the Raman spectra were measured from Δ*T* = 0 s to 240 s with the time resolution of 1 s. The photostationary state was measured for Δ*T* = 0 s. In the figure, the vibrational modes of the RSB chromophore were selectively observed due to the preresonance enhancement. Before a green-light irradiation, the unphotolyzed state exhibited the intense C=C stretch (1533 cm^−1^), C-C stretch (1168 and 1200 cm^−1^), and CH_3_ rocking (1006 cm^−1^) (17, 18). In addition, the unphotolyzed state shows the moderately strong bands assigned to the C=N stretch (1643 cm^−1^), CH in-plane bending (1270–1350 cm^−1^), and hydrogen out-of-plane (HOOP) modes (826, 879, 970 cm^−1^) (17, 18). The double peaked pattern of C-C stretching modes is characteristic of the all-*trans* form of the chromophore. This observed spectrum of the unphotolyzed state agrees with those reported previously (5, 6, 9, 19). Then, after the irradiation by green light, the Raman spectra of photointermediates appeared. As shown in the figure, the photointermediate showed the C-C stretches with three peaks at 1173, ∼1200, and 1216 cm^−1^. The C=C stretch of the photointermediate was significantly downshifted relative to that of the unphotolyzed state (e.g., 1524 cm^−1^ at Δ*T* = 1 s versus 1533 cm^−1^ at unphotolyzed state). As Δ*T* increased, these spectral features showed the changes that appear to consist of two phases. The first phase corresponds to the spectral change at Δ*T* = 0 to 1 s, where the C=C stretch shifted by +4 cm^−1^ and the intensity pattern in the C-C stretching region (1150–1250 cm^−1^) slightly changed. The second phase is after Δ*T* = 1 s, as a large intensity decrease was observed for the HOOP modes and the C-C stretch at 1173 cm^−1^.

**Figure 4:**
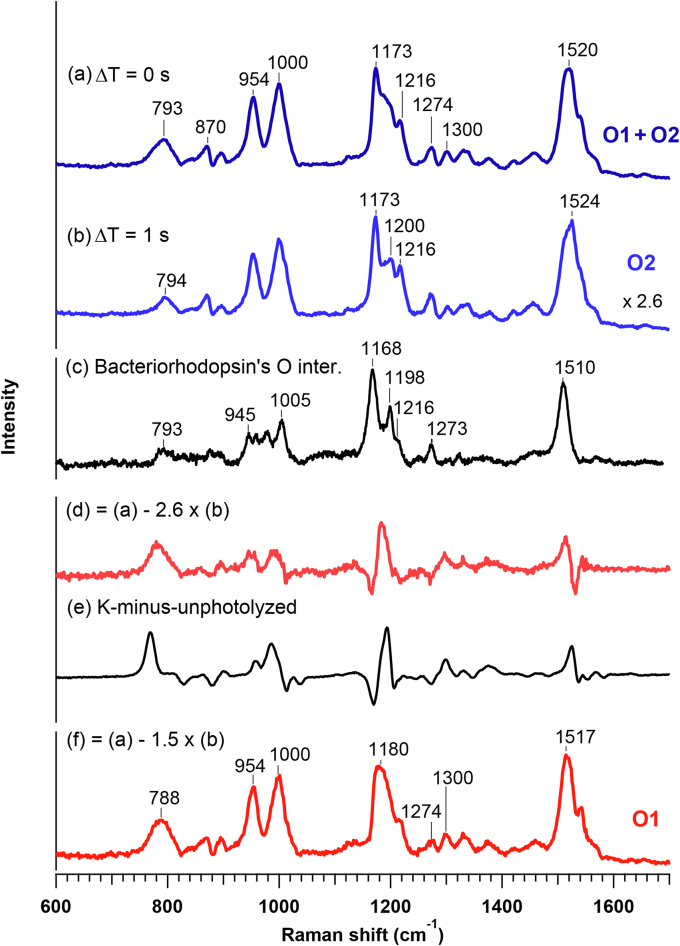
**Spectral analysis of time-resolved Raman data at 0 s and 1 s.** Time-resolved Raman spectra of *Ia*NaR at Δ*T* = 0 s (a) and 1 s (a) are compared with the Raman spectrum of the O intermediate of bacteriorhodopsin (c). (d) is the difference spectrum calculated as (a) – 2.6 × (b), and (e) is the difference spectrum between K intermediate and unphotolyzed state of *Ia*NaR. Spectral component (f) is obtained by (a) – 1.5 × (b). *Ia*NaR, NaR from *Indibacter alkaliphilus*.

In Fig. 4, we provide an analysis of the Raman spectra in the first phase, that is, Δ*T* = 0 and 1s (traces a and b). On the basis of the time-resolved absorption data (Fig. 2), this time region corresponds to the O1 → O2 process. The spectra at 0 and 1 s are characterized by the downshifted C=C stretch, the triple-peaked C-C stretches, and the intense HOOP modes. First, the C=C stretching frequency (ν_C=C_) is known to correlate with the maximum absorption wavelength (*λ*_max_); +1 cm^−1^ shift of ν_C=C_ generally corresponds to −4 nm shift of *λ*_max_ (20, 21, 22, 23). Thus, the downshifted C=C stretch is consistent with the redshift of the absorption band at 0 and 1 s. Next, the intensity pattern of the C-C stretch is sensitive to the geometry of the polyene chain (17, 18, 24). The C-C stretching bands have three peaks at 1173, ∼1200, and 1216 cm^−1^. For purposes of comparison, we measured the O intermediate of bacteriorhodopsin (BR) and plotted its spectrum in the figure (trace c). As seen, the O intermediate of BR shows the C-C stretches whose intensity patterns are very similar to the time-resolved Raman spectrum of *Ia*NaR, especially at Δ*T* = 1 s. The O intermediate of BR has the all-*trans* chromophore (25). This indicates that the all-*trans* chromophore makes a major contribution in the time-resolved spectrum at 1 s. Moreover, the intense HOOP modes were observed at 794 and 954 cm^−1^. These modes have been assigned to the HOOP vibrations of C14H and HC11=C12H (17, 18), the intensities of which are the measures for the degree of chromophore distortions (26, 27) about C11=C12 and C13=C14. Therefore, the all-*trans* RSB chromophore is significantly distorted.

As compared to the Raman spectrum at 1 s, the spectrum at 0 s is different. The C=C stretch is observed at 1520 cm^−1^ at 0 s. This C=C stretching frequency is downshifted by 4 cm^−1^ relative to that at 1 s. The HOOP intensity at ∼793 cm^−1^ is clearly larger at 0 s. Furthermore, the intensity patterns in the C-C stretching region are different between 0 and 1 s, as the peak at ∼1200 cm^−1^ is less clear at 0 s. To highlight these differences, we plotted the difference spectrum calculated as *s*_0s_ – 2.6 × s_1s_ (trace d), where *s*_0s_ and *s*_1s_ denote the spectra at Δ*T* = 0 s and 1 s, respectively, and the subtraction parameter 2.6 is chosen to equalize the intensities of the C=C stretch. This difference spectrum clearly represents the significant structural change from Δ*T* = 0 to 1 s. In Fig. 4, we also plotted the difference spectrum between the K intermediate and the unphotolyzed state of *Ia*NaR measured at 80 K (5) (K-minus-unphotolyzed, trace e) for comparison. The K-minus-unphotolyzed spectrum represents the spectral change from the all-*trans* to 13-*cis* chromophore. Importantly, it shows a close similarity with the difference spectrum between Δ*T* = 0 s and 1 s. This indicates that the spectral change from Δ*T* = 0 s to 1 s is caused by isomerization of the chromophore. Actually, when we take the spectral difference as *s*_0s_ – 1.5 × s_1s_ so as to subtract the intense 1173-cm^−1^ band of the all-*trans* form, the spectral component of a 13-*cis* form is obtained as trace f. The single-band C-C stretch at 1180 cm^−1^ is characteristic of the 13-*cis* chromophore, and the intense HOOP modes at 788 and 954 cm^−1^ signify the distortion of chromophore. In short, the two photointermediates have the major contributions at 0 s; one has the distorted all-*trans* chromophore, and the other has the distorted 13-*cis* form.

According to our time-resolved absorption measurements (Fig. 2), we can assign the two photointermediates observed at 0 s to O1 and O2. O1 has the distorted 13-*cis* chromophore and decays within 1 s, while O2 has the distorted all-*trans* form that appears predominantly at 1 s. The later photointermediates, N and NaR′, may contribute to the Raman spectrum at 0 s under photostationary state, but they are not clearly recognized at 0 s due likely to the relatively small preresonance enhancement.

Table 1 lists the vibrational frequencies and *λ*_max_ values characteristic of O1 and O2. In the previous time-resolved absorption study of *Ia*NaR, we estimated that the *λ*_max_ values of O1 and O2 were 596 and 570 nm, respectively, at room temperature (14). The 26 nm blueshift of *λ*_max_ is consistent with the 7 cm^−1^ upshift of ν_C=C_ from O1 to O2, considering the general correlation between ν_C=C_ and *λ*_max_ (Supporting Information, Fig. S3). We also carried out the experiment at lower temperatures (250 and 260 K) and confirmed that the intermediate, which we assigned to O1 at 268 K, was actually produced from the L-like precursor (Supporting Information, Fig. S4).

**Table 1:** *λ*_max_ values (nm) and selected vibrational frequencies (cm^−1^) of O1 and O2

Intermediate	*λ*_max_^a^	C=C str.	C-C str.	14H HOOP
O1	596	1517	∼1180	788
O2	570	1524	1168, 1200, 1216	794

a At room temperature.

**Figure 5:**
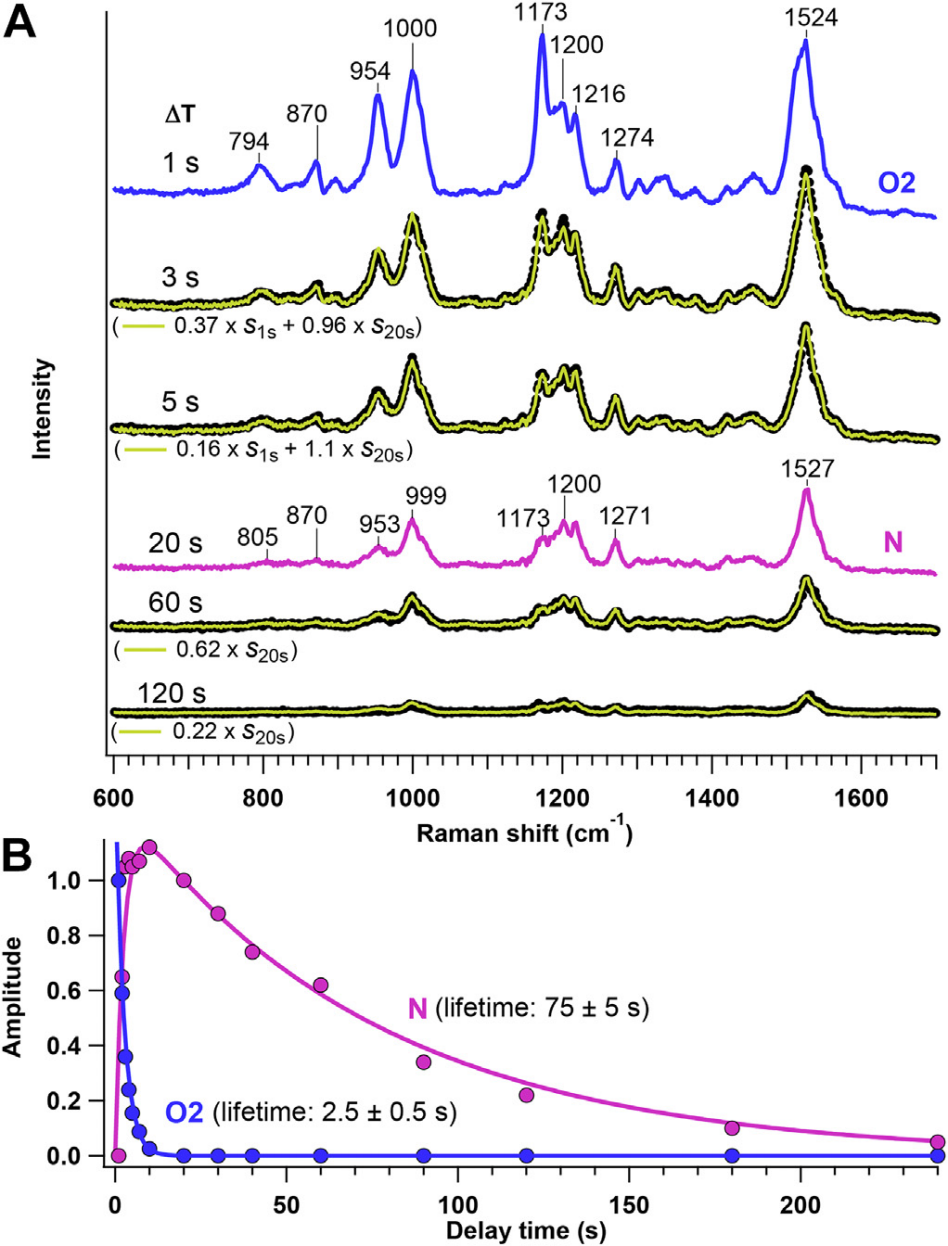
**Kinetic analysis of time-resolved Raman data of*****Ia*****NaR at 268 K.** (A), The time-resolved Raman spectra after Δ*T* = 1 s are compared with the linear combination of the spectra at Δ*T* = 1 s and 20 s, which are attributed to O2 and N, respectively. The time-resolved data at selected Δ*T*’s (3, 5, 60, and 120 s) are shown with *black dots* and compared with the linear combination expressed by *a* × *s*_1s_+*b* × *s*_20s_ (*yellow line*): *s*_1s_ and *s*_20s_ denote the spectra at Δ*T* = 1 s and 20 s, respectively, and *a* and *b* represent their amplitudes. *a* and *b* are used for the kinetic analysis in (B), where lifetimes of O2 and N are determined by curve fitting. *Ia*NaR, NaR from *Indibacter alkaliphilus*.

Then, we examine the time-resolved Raman spectra in the second phase (Δ*T* = 1–240 s) in Fig. 5. As described above, the spectral feature at 1 s is attributable to the distorted all-*trans* form of O2. With the increase of Δ*T*, however, this spectral feature disappeared in several seconds. Specifically, the intensity of the C-C stretch at 1173 cm^−1^ shows the large decrease, and the C-C stretching region at 20 s consists of the shoulder at 1173 cm^−1^ and the main bands at ∼1200 cm^−1^ (Fig. 3). This intensity pattern at 20 s is still characteristic of the all-*trans* chromophore (28) but the loss of the intense 1173 cm^−1^ mode is indicative of a structural change. Indeed, the relative intensities of the HOOP modes decreased in 20 s, representing the reduced distortion of the all-*trans* chromophore. As shown in Fig. 5*A*, the time-resolved spectra from 1 to 20 s were reproduced by the linear combination of the data at 1 and 20 s. No spectral change after 20 s was observed within experimental uncertainty. Therefore, the time-dependent spectral change is explained by the population change of O2 and the subsequent intermediate. The kinetic analysis in Fig. 5*B* showed that the lifetime of O2 is 2.5 s and the subsequent intermediate rises with the O2 decay. This intermediate produced in 20 s is thus assignable to N. The lifetime of N is estimated as 75 s. N and NaR′ were spectrally indistinguishable.

## Discussion

From the time-resolved Raman spectra of *Ia*NaR at 268 K, the multiple redshifted intermediates (O1, O2, and N), which have distinct chromophore structures, are revealed. The vibrational spectra of these photointermediates showed that O1 has the distorted 13-*cis* chromophore, O2 has the distorted all-*trans* chromophore, and N has the relaxed all-*trans* form. This result also points out that the structures of the O intermediates reported to date should be reconsidered because a single O intermediate was assumed in most of previous studies of NaRs. The early light-induced FTIR measurement of an NaR from *Gillisia limnaea* revealed the O intermediate with all-*trans* chromophore under photostationary state (10), and the spectrum was close to that of the O intermediate of bacteriorhodopsin. A recent NMR study of KR2 also reported such an O intermediate that has an all-*trans* form (11). This state is now seen as the O2 intermediate. On the other hand, a time-resolved FTIR study of KR2 and NdR2 (NaR from *Nonlabens dokdonensis*) reported an O intermediate with a distorted 13-*cis* chromophore in the millisecond region (8). This state is assignable to O1. Recently, time-resolved Raman spectroscopy of KR2 observed the photointermediate in the millisecond region, which exhibited the broad C=C stretch (at 1524 cm^−1^) and the multi-peaked C-C stretches (at 1172, 1185, 1197, and 1218 cm^−1^) (9). Although this spectrum was assigned to the single O intermediate containing a 13-*cis* chromophore, we suspect that the spectrum arose from a mixture of O1 and O2.

Concerning the X-ray crystallographic studies, two groups independently reported the different crystal structures of the O intermediate of KR2 (12, 13). One structure (PDB ID: 6XYT) was solved by Kovalev *et al*. and has the all-*trans* chromophore with Na^+^ bound to D115 (D116 in KR2) near the active site (13). The other (PDB ID: 6TK2) was solved by Skopintsev *et al*. and has the 13-*cis* chromophore with Na^+^ bound to D250 (D251 in KR2) (12). Recent QM/MM (quantum mechanical/molecular mechanical) calculations showed that the Na^+^ binding to these sites causes the significant redshift of the absorption band of the O intermediates (29). We consider that either O1 or O2 was almost selectively observed in these studies. Normally, it would be natural to assume that Na^+^ moves to an extracellular site from O1 to O2 transition (29, 30). Therefore, in terms of the Na^+^-binding position, the one structure (PDB ID: 6XYT) may correspond to O1 and the other (PDB ID: 6TK2) to O2. If such is the case, however, the puzzling point is that the chromophore structures are inconsistent with our observation. As both groups discussed the coexistence of all-*trans* and 13-*cis* forms (12, 13), the chromophore structures in these crystallographic studies need to be carefully interpreted.

**Figure 6:**
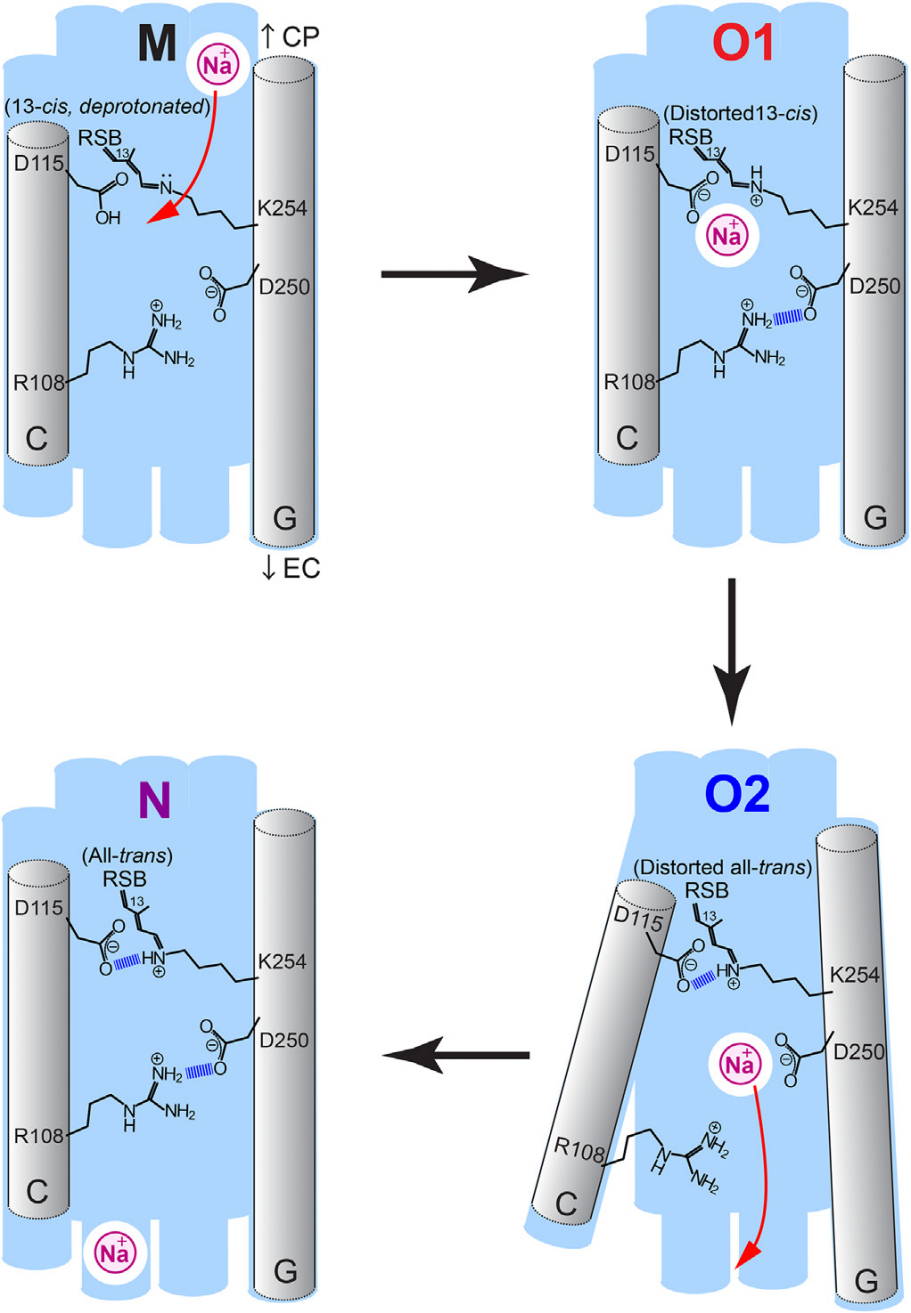
**Molecular model of Na**^**+**^**translocation mediated by O intermediates in *Ia*NaR.** CP, cytoplasm; EC, extracellular side; and RSB, retinal Schiff base. The amino acid sequence numbers in *Ia*NaR are used. *Ia*NaR, NaR from *Indibacter alkaliphilus*.

The chromophore structure of O1 and O2 can provide crucial insight about the Na^+^ pumping mediated by the O intermediates. Our time-resolved absorption and membrane potential measurements revealed that O1 is responsible for Na^+^ uptake, O2 is responsible for Na^+^ release, and the O1 → O2 transition was irreversible (7, 14). The present Raman study indicates that the reisomerization of the RSB chromophore acts as the irreversible switch between the Na^+^ uptake and release states. During the Na^+^ pumping through O1 and O2, an Na^+^-binding site inside the protein needs to be accessible from the cytoplasmic side only in the uptake process (L/M → O1), and the binding site then must be open to the extracellular side in the release process (O2 → N). In Fig. 6, we propose a molecular model of this Na^+^ transport for *Ia*NaR. In this model, Na^+^ passes in close proximity to the neutral deprotonated chromophore in the M/L state, and it binds to the D115 (D116 in KR2) in O1. For this binding site, we referred to the crystal structure (PDB ID: 6XYT) solved by Kovalev *et al*. (13) (*vide supra*). The protonated 13-*cis* chromophore in O1 is considered to flip the positively charged Schiff base moiety opposite to D115. Thus, the formation of the electrostatic pair of Na^+^ and D115 is temporarily allowed. In O1, however, the protonated Schiff base cannot be stabilized due to the lack of efficient electrostatic interaction with the counter ion. This situation may drive the isomerization of the chromophore to interact with D115 and produce O2. In forming O2, the all-*trans* chromophore points the Schiff base moiety toward D115, which destabilizes the Na^+^ binding to D115 and causes Na^+^ to move to the D250 site (D251 in KR2). We referred to the crystal structure (PDB ID: 6TK2) solved by Skopintsev *et al*. (12) (*vide supra*) for the interaction between Na^+^ and D250. When Na^+^ binds to D250, the ionic hydrogen bond (or salt bridge) of D250-R108 would be transiently broken. This would make the binding site open to the extracellular side. Then, Na^+^ is finally pumped to the outside of the membrane in the O2 → N step. In the N intermediate, we assume that the R108-D250 salt bridge is reformed to prevent the backflow of Na^+^. This protein structural change may be associated with the relaxation of the chromophore conformation.

## Conclusion

We used time-resolved Raman spectroscopy to study the structures of O intermediates of *Ia*NaR at 268 K. The present time-resolved Raman experiment under the preresonance condition allowed us to observe high quality vibrational spectra of three intermediates. These intermediates have distinct chromophore structures, and they were attributed to O1, O2, and N intermediates. O1 has the distorted 13-*cis* chromophore while O2 has the distorted all-*trans* form. The subsequent N has the relaxed all-*trans* chromophore. This study indicated that the thermal reisomerization of the chromophore occurs during the O1 → O2 process, where O1 is responsible for Na^+^-uptake and O2 is responsible for Na^+^-release. The irreversibility of the O1 → O2 step, which is key for the unidirectional Na^+^ pump, is also realized by the reisomerization of the RSB chromophore. The isomerization of the RSB chromophore is considered to drastically change the electrostatic interactions in the active site to translocate Na^+^. The O2 → N process is associated with the chromophore structural relaxation. This structural relaxation of the chromophore should involve the protein conformational change to prevent the backflow of Na^+^.

## Experimental procedures

The Raman spectra were measured using a continuous wave laser (08-NLD(M), Cobolt) operating at 785 nm. The laser power at the sample was 350 mW. The back scattered light from the sample was collected and dispersed by a 30 cm polychromator with a 600 groove/mm grating (Acton SP-2300; Princeton Instruments). The spectrum was then measured by a charge-coupled device detector (Pixis 256E; Princeton Instruments). Using this setup, the preresonance Raman spectra of *Ia*NaR were obtained with a resolution of 8.5 cm^−1^. The time-resolved Raman measurement of *Ia*NaR was carried out at 268 K. At this temperature, the photocycle of *Ia*NaR was slowed enough that the lifetimes of photointermediates were extended for a duration on the order of seconds. To perform the time-resolved measurement, we irradiated the sample with a 532 nm light-emitting diode (LED) (0.38 mW; thorlabs) for 1 s, and the Raman spectra were collected with an exposure time of 1 s at the selected time delays (Δ*T*’s). The time resolution was therefore 1 s. The photostationary state under continuous irradiation was measured for Δ*T* = 0. The Raman spectra of photointermediates were obtained by subtracting the unphotolyzed component from the spectra after light irradiation (Supporting Information, Figs. S1 and S2). We also checked the LED-irradiation power dependence of the Raman spectrum and confirmed that there was no photoproduct due to a multiphoton process (Supporting Information, Figs. S1 and S2). For a comparison, we prepared a BR sample using a 10 mM citrate buffer solution (pH 4.0) and measured the O intermediate under a photostationary state at room temperature (31).

The time-resolved absorption measurement was also performed at 268 K. We used a white LED (Edison) as the probe light. After the sample was irradiated by the 532-nm LED for 1 s, the probe light transmitted through the sample was dispersed by a 30 cm polychromator (with a 300 groove/mm grating), and the spectrum was measured by the charge-coupled device detector with an exposure time of 1 s at the selected Δ*T*’s. The photostationary state was measured for Δ*T* = 0. The light-induced absorbance change was calculated from the spectra of transmitted lights before and after the photoirradiation, and the time-resolved absorption spectra were obtained.

The *Ia*NaR sample was prepared as reported previously (6). Briefly, *Ia*NaR, having a C-terminal histidine tag, was expressed in *Escherichia coli* strain BL21 by using the pKA001 vector which contains *Ia*NaR gene (GenBank accession number: EOZ93469) under the lacUV5 promoter. The cells were disrupted by sonication and the membrane fragments were collected by ultracentrifugation. After solubilization with the detergent *n*-dodecyl *β*-D-maltopyranoside, *Ia*NaR was purified using a nickel–nitrilotriacetic acid agarose column. The purified sample was reconstituted in Egg-PC liposome at a protein: lipid molar ratio of 1:50 and stored in a 50 mM Tris buffer solution (pH 8.0 with 0.4 M NaCl). We used a film-like sample for the time-resolved Raman and absorption measurements at 268 K. After rinsing the *Ia*NaR sample with 2 mM Tris buffer solution (pH 8.0 with 3 mM NaCl), ∼1 μl drop of the suspending solution was dried on a glass plate and this drying was repeated about 7 times to concentrate the sample on the plate. Then, a water drop (∼1 μl) was added on the film-like sample to humidify it. The sample was sealed with an O ring and another glass plate and set in the liquid-nitrogen–cooled cryostat (Optstat DN2; Oxford Instruments) for the spectroscopic measurements.

## Data availability

All the data supporting the findings of this study are available within the article and the supporting information.

## Supporting information

This article contains supporting information (6, 17, 18, 25, 28, 32, 33, 34, 35, 36, 37).

## Conflicts of interest

The authors declare that they have no conflicts of interest with the contents of this article.
