# Supplementary material for: Reisomerization of retinal represents a molecular switch mediating Na+ uptake and release by a bacterial sodium-pumping rhodopsin
Source: J Biol Chem. 2022 Aug 11;298(9):102366. doi: 10.1016/j.jbc.2022.102366 (PMC9483557; doi:10.1016/j.jbc.2022.102366)
Supplement: Supporting Information [file mmc1.pdf]

Supporting Information

Reisomerization of retinal represents a molecular switch mediating Na^+ uptake and release by a bacterial sodium-pumping rhodopsin

Tomotsumi Fujisawa[1]*, Kouta Kinoue[1], Ryouhei Seike[1], Takashi Kikukawa[2], Masashi Unno[1]*

[1] *Department of Chemistry and Applied Chemistry, Faculty of Science and Engineering, Saga University, Saga 840-8502, Japan*

[2] *Faculty of Advanced Life Science, Hokkaido University, Sapporo 060-0810, Japan*

*Correspondence: Tomotsumi Fujisawa, Masashi Unno
Email: tfuji@cc.saga-u.ac.jp, unno@cc.saga-u.ac.jp

Contents

1. Procedure to extract time-resolved Raman spectra of photointermediates

2. Correlation between $\nu_{C=C}$ and λ_{max} for photointermediates of *Ia*NaR

3. Presence of L-like intermediate at 260 K and 250 K

1. Procedure to extract time-resolved Raman spectra of photointermediates

In our time-resolved Raman measurement, *Ia*NaR sample was pumped by green LED (532 nm) for 1 second, and then the Raman spectra were measured by 785-nm probe at selected time delays (ΔT's) with the exposure time of 1 second. To obtain the Raman spectra of photointermediates, we measured the Raman spectrum of unphotolyzed state (probe-only spectrum) as well as that with 532-nm pump (pump+probe spectrum). Figure S1 shows the pump+probe spectrum (trace a) and the probe-only spectrum (trace b) at $\Delta T = 1$ s. The spectrum of photointermediate(s) is obtained after subtracting the contribution of the unphotolyzed state as (a) – (b) × 0.9. In the subtraction, the unphotolyzed component was well estimated by using the band intensity at 826 cm^{-1}. We also confirmed that no pump-power dependence was observed up to 0.38 mW.

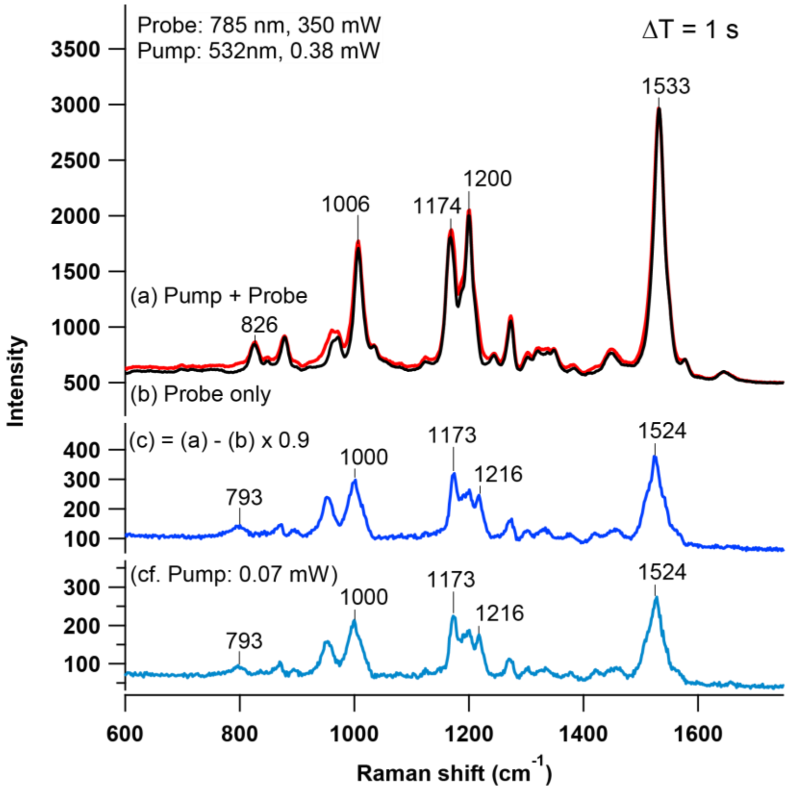

Figure S1. Time-resolved Raman spectrum measured using 0.38 mW photoirradiation at $\Delta T = 1$ s. (a) Pump+Probe, (b) Probe-only, (c) spectrum of photointermediate. Trace (c) is compared with the spectrum obtained using 0.07 mW photoirradiation.

For photostationary state, i.e., $\Delta T = 0$ s, we additionally measured the spectrum of the fluorescence background due to 532-nm pump (pump-only spectrum). Figure S2 shows how we obtained the spectrum of photointermediate(s) at $\Delta T = 0$ s. Firstly, we remove the fluorescence background by subtracting the pump-only spectrum (trace b) from the pump+probe spectrum (trace a) so as to obtain the spectrum without fluorescence background (trace c). Then, the contribution of unphotolyzed-state

spectrum (trace d) was subtracted from the trace c as (c) − (d) × 0.83 No critical pump-power dependence was observed up to 0.38 mW under photostationary condition.

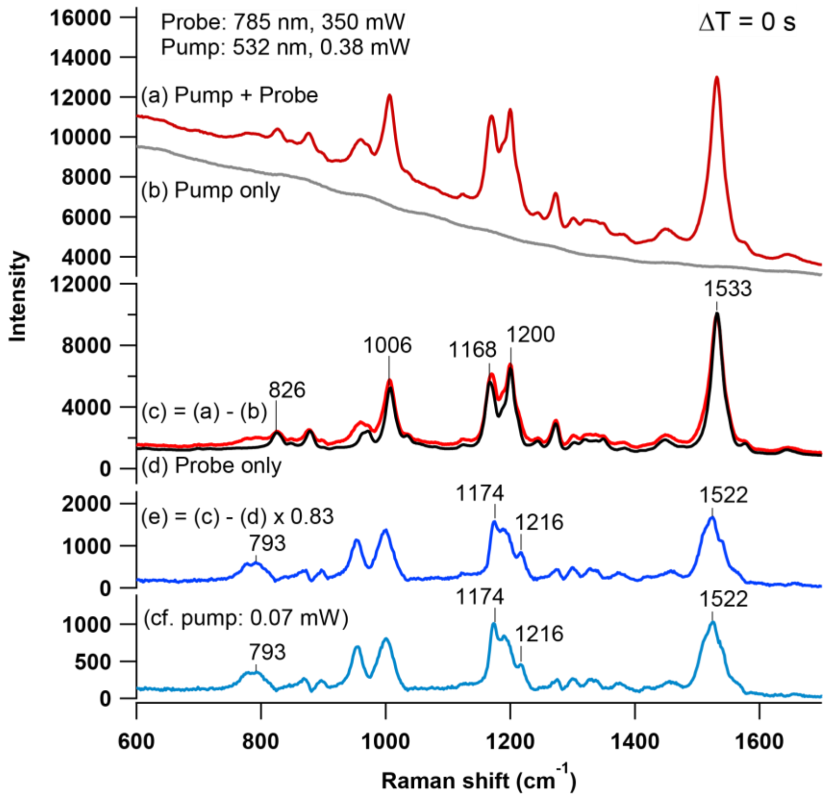

Figure S2. Time-resolved Raman spectrum measured using 0.38 mW photoirradiation at Δ*T* = 0 s. (a) Pump+Probe, (b) Pump-only, (c) spectrum after subtracting (b) from (a), (d) Probe-only, (e) spectrum of photointermediate. Trace (e) is compared with the spectrum obtained using 0.07 mW photoirradiation.

2. Correlation between $\nu_{C=C}$ and λ_{max} for photointermediates of *Ia*NaR

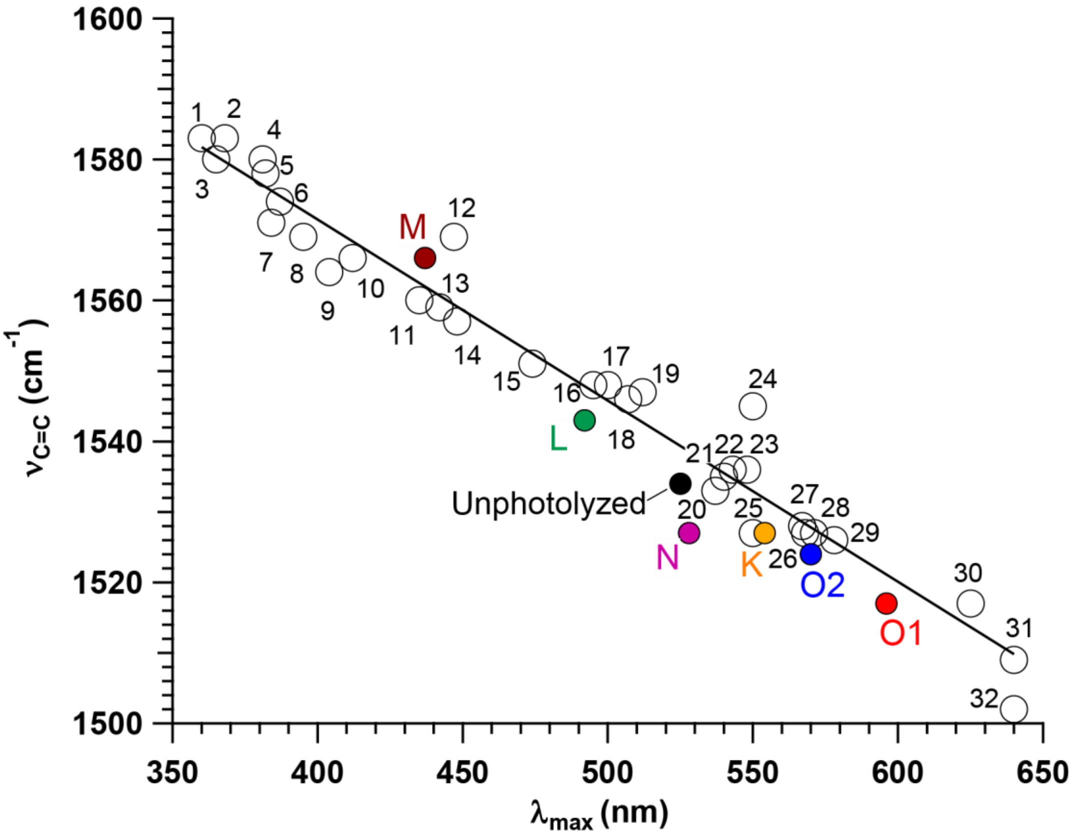

Figure S3. Correlation between $\nu_{C=C}$ and λ_{max} (1-11). Open circle: (1) all-*trans* retinal + *n*-$C_6H_{13}NH_2$ in C_6H_{14}, (2) all-*trans* retinal + *n*-$C_5H_{13}NH_2$ in $C_8H_{17}OH$, (3) all-*trans* retinal + *n*-$C_6H_{13}NH_2$ in C_6H_6, (4) all-*trans* retinal in C_6H_6, (5) all-*trans* retinal in CCl_4, (6) all-*trans* retinal in $C_8H_{17}OH$, (7) all-*trans* retinal + aniline in *n*-C_6H_{14}, (8) all-*trans* retinal + aniline in C_2H_5OH, (9) all-*trans* retinal + *p*-bromoaniline in C_2H_5OH, (10) bacteriorhodopsin's M intermediate, (11) all-*trans* retinal + hexylamine in *n*-C_6H_{14}, (12) toad rhodopsin, (13) all-*trans* retinal + hexylamine in $C_8H_{12}OH$, (14) all-*trans* retinal + hexylamine in C_2H_5OH, (15) gecko rodopsin, (16) bovine isorhodopsin, (17) sensory rhodopsin II from *Natronomonas pharaonis*, (18) bovine rhodopsin, (19) all-*trans* retinal + *p*-bromoaniline in acidified C_2H_5OH, (20) human green cone, (21) *Gloeobacter* rhodopsin, (22) bovine bathorhodopsin, (23) bacteriorhodopsin in dark-adapted state, (24) bacteriorhodopsin's L intermediate, (25) isoiodopsin, (26) bacteriorhodopsin in light-adapted state, (27) human red cone opsin, (28) iodopsin, (29) halorhodopsin, (30) bacteriorhodopsin's K intermediate, (31) bacteriorhodopsin's O intermediate, (32) bathoiodopsin. Closed circle: (black) Unphotolyzed state, (yellow) K intermediate, (green) L intermediate, (brown) M intermediate of *Ia*NaR, (blue) photointermediate of *Ia*NaR assigned to O2 in this study, (red) photointermediate of *Ia*NaR assigned to O1 in this study, (purple) photointermediate of *Ia*NaR assigned to N in this study.

3. Presence of L-like intermediate at 260 K and 250 K

As in Figure 4 (main text), we obtained the Raman spectrum of O1 at 268 K from the spectral difference calculated as $\boldsymbol{s}_{0\text{s}} - f \times \boldsymbol{s}_{1\text{s}}$, where $\boldsymbol{s}_{0\text{s}}$ and $\boldsymbol{s}_{1\text{s}}$ denote the spectra at $\Delta T = 0$ s and 1 s respectively, and 1.5 is used for the subtraction parameter *f*. In Figure S4, we found that the spectrum thus obtained clearly changed as the temperature decreased down to 250 K. As seen from the red spectra in the figure, a sharp HOOP mode appeared at 774 cm^{-1} as temperature decreases. The single-band C-C stretch at 260 K and 250 K indicates that chromophore is 13-*cis* form, but the frequency showed a upshift. Particularly, the C=C stretching frequency ($\nu_{C=C}$) showed the significant upshift and the $\nu_{C=C}$ value (1536 cm^{-1}) at 250 K was higher than that of the unphotolyzed state (1534 cm^{-1}). These spectral features are attributed to the L-like intermediate as the precusor of O1.

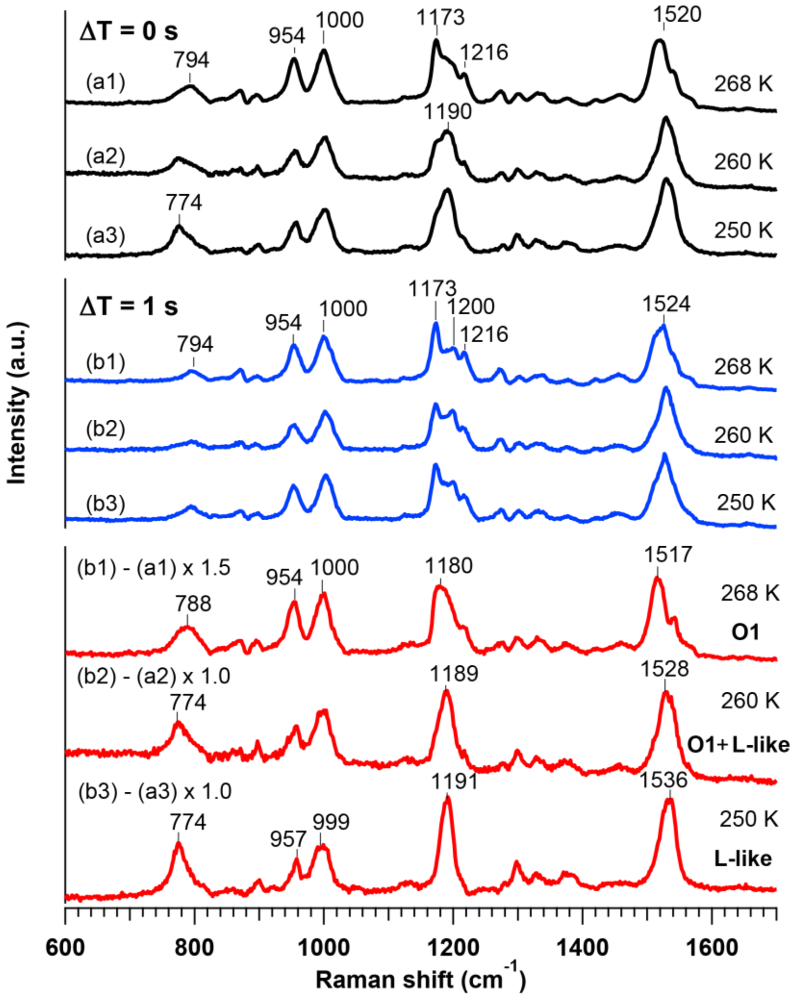

Figure S4. Temperature dependence of time-resolved Raman spectra at $\Delta T = 0$ and 1 s: (black) spectra at $\Delta T = 0$ s measured at 268, 260, and 250 K; (blue) spectra at $\Delta T = 1$ s measured at 268, 260, and 250 K; (red) spectral difference obtained as $\boldsymbol{s}_{0\text{s}} - f \times \boldsymbol{s}_{1\text{s}}$, where $\boldsymbol{s}_{0\text{s}}$ and $\boldsymbol{s}_{1\text{s}}$ denote the spectra at $\Delta T = 0$ s and 1 s respectively, and *f* is the subtraction parameter.

References

1. Smith, S. O., Marvin, M. J., Bogomolni, R. A., and Mathies, R. A. (1984) Structure of the retinal chromophore in the hR578 form of halorhodopsin. *J. Biol. Chem.* **259**, 12326-12329
2. Iizuka, A., Kajimoto, K., Fujisawa, T., Tsukamoto, T., Aizawa, T., Kamo, N., Jung, K. H., Unno, M., Demura, M., and Kikukawa, T. (2019) Functional importance of the oligomer formation of the cyanobacterial H^+ pump *Gloeobacter* rhodopsin. *Sci Rep* **9**, 10711
3. Smith, S. O., Pardoen, J. A., Lugtenburg, J., and Mathies, R. A. (1987) Vibrational analysis of the 13-*cis*-retinal chromophore in dark-adapted bacteriorhodopsin. *J. Phys. Chem.* **91**, 804-819
4. Smith, S. O., Braiman, M. S., Myers, A. B., Pardoen, J. A., Courtin, J. M. L., Winkel, C., Lugtenburg, J., and Mathies, R. A. (1987) Vibrational analysis of the all-trans-retinal chromophore in light-adapted bacteriorhodopsin. *J. Am. Chem. Soc.* **109**, 3108-3125
5. Smith, S. O., Myers, A. B., Pardoen, J. A., Winkel, C., Mulder, P. P., Lugtenburg, J., and Mathies, R. (1984) Determination of retinal Schiff base configuration in bacteriorhodopsin. *Proc. Natl. Acad. Sci. U.S.A.* **81**, 2055-2059
6. Smith, S. O., Pardoen, J. A., Mulder, P. P. J., Curry, B., Lugtenburg, J., and Mathies, R. (1983) Chromophore structure in bacteriorhodopsin's O_{640} photointermediate. *Biochemistry* **22**, 6141-6148
7. Heyde, M. E., Gill, D., Kilponen, R. G., and Rimai, L. (1971) Raman spectra of Schiff bases of retinal (models of visual photoreceptors). *J. Am. Chem. Soc.* **93**, 6776-6780
8. Kochendoerfer, G. G., Wang, Z., Oprian, D. D., and Mathies, R. A. (1997) Resonance Raman examination of the wavelength regulation mechanism in human visual pigments. *Biochemistry* **36**, 6577-6587
9. Braiman, M., and Mathies, R. (1980) Resonance Raman evidence for an all-trans to 13-*cis* isomerization in the proton-pumping cycle of bacteriorhodopsin. *Biochemistry* **19**, 5421-5428
10. Gellini, C., Luttenberg, B., Sydor, J., Engelhard, M., and Hildebrandt, P. (2000) Resonance Raman spectroscopy of sensory rhodopsin II from *Natronobacterium pharaonis*. *FEBS Lett.* **472**, 263-266
11. Kajimoto, K., Kikukawa, T., Nakashima, H., Yamaryo, H., Saito, Y., Fujisawa, T., Demura, M., and Unno, M. (2017) Transient resonance Raman spectroscopy of a light-driven sodium-ion-pump rhodopsin from *Indibacter alkaliphilus*. *J. Phys. Chem. B* **121**, 4431-4437
